# A Cl^−^ Hinge for Cyclen Macrocycles: Ionic Interactions and Tweezer–Like Complexes

**DOI:** 10.3389/fchem.2019.00143

**Published:** 2019-03-22

**Authors:** Juan Ramón Avilés–Moreno, Giel Berden, Jos Oomens, Bruno Martínez–Haya

**Affiliations:** ^1^Department of Physical, Chemical and Natural Systems, Universidad Pablo de Olavide, Seville, Spain; ^2^FELIX Laboratory, Institute for Molecules and Materials, Radboud University, Nijmegen, Netherlands

**Keywords:** molecular recognition, macrocycles, cyclen, chloride, infrared spectroscopy

## Abstract

The supramolecular networks derived from the complexation of polyazamacrocycles with halide anions constitute fundamental building blocks of a broad range of modern materials. This study provides insights into the conformational framework that supports the binding of protonated cyclen macrocyles (1,4,7,10-Tetraazacyclododecane) by chloride anions through NH^δ+^···Cl^−^ interactions. The isolated complex comprised of two cyclen hosts linked by one Cl^−^ anion is characterized by means of infrared action spectroscopy and ion mobility mass spectrometry, in combination with quantum chemical computations. The Cl^−^ anion is found to act as a hinge that bridges the protonated ${\text{NH}}_{2}^{+}$ moieties of the two macrocycles leading to a molecular tweezer configuration. Different types of conformations emerge, depending on whether the trimer adopts an open arrangement, with significant freedom for internal rotation of the cyclen moieties, or it locks in a folded conformation with intermolecular H-bonds between the two cyclen backbones. The ion mobility collision cross section supports that folded configurations of the complex are dominant under isolated conditions in the gas phase. The IRMPD spectroscopy experiments suggest that two qualitatively different families of folded conformations coexist at room temperature, featuring either peripheral or inner positions of the anion with respect to the macrocycle cavities, These findings should have implications in the growth of extended networks in the nanoscale and in sensing applications.

## 1. Introduction

The supramolecular complexes of polyazamacrocycles with halide anions conform intermediate arrangements in the synthesis of a broad range of modern nanostructured materials, with typically catalytic or ion-exchange activity (Alper et al., 1991; Ilioudis and Steed, 2001; Warden et al., 2004a; Mateus et al., 2010; Park et al., 2012; Wenzel et al., 2012; Lee et al., 2013, 2015; Evans and Beer, 2014; Busschaert et al., 2015). The rationalization of the mechanisms of growth of specific molecular networks is challenging and benefits from the fundamental insights and the validation of computational methods gained from the study of precursor macrocycle–anion clusters. The conformational landscape of these clusters can actually become quite complex, depending on the size of the macrocycle and on the degree of protonation of its amine groups (Boudon et al., 1991; Ilioudis and Steed, 2001; Warden et al., 2004a,b; Wichmann et al., 2006; Mateus et al., 2010; Wang et al., 2016). The aim of this work is to contribute to the understanding of the anionic supramolecular chemistry of azamacrocycles through the characterization of benchmark aggregates involving the binding of Cl^−^ to protonated cyclen (tetraazacyclododecane).

Previous works have investigated the recognition and binding of anions by azamacrocyles and related receptors employing condensed-phase methods, typically UV-vis absorption and fluorescence, NMR spectroscopy, or crystallography techniques (Ilioudis and Steed, 2001; Wichmann et al., 2006; Wenzel et al., 2012; Evans and Beer, 2014; Busschaert et al., 2015). Our investigation is rather based on a systematic investigation of complexes of well-defined stoichiometry under isolated conditions (Rijs and Oomens, 2015). On the one hand, ion mobility mass spectrometry (Jurado-Campos et al., 2018) is employed to obtain a measure of the conformational shape of the complexes. On the other hand, action infrared action spectroscopy (Polfer and Oomens, 2009) serves to elucidate the vibrational modes of the complex after mass selection and storage in an ion trap. The two experimental approaches provide complementary information: where ion mobility probes global structure (overall shape), infrared action spectroscopy is sensitive to the effect that conformations have on the local structure of the complex (atomic interactions and bond strengths). The experiments are analyzed in the light of quantum chemical modeling of the conformational and vibrational features of the isolated molecular systems. In a recent investigation, we employed this methodology to characterize protonated cyclen and provide insights into the structure and intramolecular interactions in the isolated macrocycle (Avilés-Moreno et al., 2018). The present study focuses on supramolecular features that should be relevant to the modeling of azamacrocycle networks, such as the preferred coordination arrangement sustained by the Cl^−^ anions and the relative stability of packed sandwich-like configurations vs. open chain-like arrangements. Intermolecular and intramolecular proton bonding networks are analyzed and their implication in the structure of the complex is discussed. Despite the challenges imposed by proton interactions to the accurate description of the system, it is shown that the interrelation between experimental spectroscopy and computations provides insights into fundamental supramolecular features of azamacrocycle/halide frameworks.

## 2. Methods

### 2.1. IRMPD Spectroscopy

The infrared multiple photon dissociation (IRMPD) spectroscopy experiments were carried out at the Fourier Transform Ion Cyclotron Resonance mass spectrometry (FT–ICR) beamline of the free electron laser FELIX.^1^ IRMPD is a type of action spectroscopy, based on the detection of photofragments produced by the sequential absorption of infrared photons at resonant wavelengths (Polfer and Oomens, 2009).

The ionic complexes were produced by means of electrospray ionization of a 1 mM solution of cyclen (97% purity) and KCl salt (99.9 % purity) in 1:1 water/methanol, acidified with diluted HCl. The resulting product ions were pulse injected into the ICR cell for storage at room temperature. The mass spectrum displayed strong signals at the nominal masses *m*/*z* = 381/383, which were assigned to the (cyclen·H^+^)_2_·Cl^−^ ions (i.e., two protonated cyclen macrocycles bound to a chloride anion). This identification was based on the relative intensities of the isotopic peaks (^35^Cl/^37^Cl ~ 3:1, indicating the presence of one chloride anion) and on the corresponding exact masses (*m*/*z* = 381.321/383.318) as determined in separate experiments in which the sample solution was electrosprayed into a high resolution orbitrap mass spectrometer (model Q-Exactive Focus, Thermo Scientific, mass resolution M/ΔM= 70000).

For the IRMPD spectroscopy experiments, the mass isolated ions were irradiated with 8 free-electron laser infrared macro–pulses. Each macro–pulse is approximately 5 microsecond long, has an energy of about 35 mJ, and consists of a train of micro–pulses with a repetition frequency of 1 GHz. The nominal spectral bandwidth of the radiation amounts to 0.5% of the central wavelength. The dominant IRMPD cationic fragment detected in the present experiment was protonated cyclen (*m*/*z* = 173). The IRMPD spectrum was constructed by monitoring the total fragment yield as a function of the wavenumber of the radiation, with linear corrections of the ion yield to account for changes in laser pulse power during scans.

### 2.2. Ion Mobility Spectrometry

Ion mobility of the isolated (cyclen·H^+^)_2_·Cl^−^ complexes with *m*/*z* = 381 was performed in two separate commercial equipments, namely a Bruker TIMS-TOF mass spectrometer and a Waters Vion IMS QTOF mass spectrometer, with N_2_ as buffer collision gas. Several replicates were run leading to a data dispersion of less than 0.1% for the average cross section in each equipment. The resulting room temperature N_2_ collision cross sections of the ions were 196.4 and 200.3 Å^2^, respectively. Such difference can be attributed to the technical specificities of the two ion mobility spectrometers, resulting in slightly different calibrations of the cross sections. The value 198 ± 2 Å^2^ will be assumed in the present work.

### 2.3. Quantum Chemistry Calculations

*Ab initio* MP2 quantum chemical computations were employed to characterize the low energy conformations of the (cyclen·H^+^)_2_·Cl^−^ ions. An initial ensemble of candidate molecular structures was produced by means of simulated annealing with the universal force field. Additional initial conformations were inspired by the NMR and crystallography data available for related azamacrocyle polychloride complexes (Boudon et al., 1991; Ilioudis and Steed, 2001; Warden et al., 2004b). Independent computations were run for the (cyclen·H^+^)·Cl^−^ subunit to include its most stable coordination arrangements as building blocks in potential seeding structures of the full trimeric complex.

About one hundred non-redundant structures of the complexes were produced, which were initially optimized with density functional theory at the B3LYP-D3 level (B3LYP functional with Grimme's D3 dispersion correction) with the 6-311++G(d,p) basis set. The around fifty most stable conformers were subsequently reoptimized at the MP2/6-311++G(d,p) level. Relative vibrational zero-point corrected electronic energies (Δ*E*_*zp*_) were considered to rank the conformations. Natural bond orbital (NBO) analysis (Foster and Weinhold, 1980) was employed for a detailed characterization of the ionic interactions that contribute to the conformational stabilization of the chloride-cyclen complexes.

For comparison with the ion mobility measurements, room temperature collision cross sections with N_2_ were computed for the low energy MP2 conformers of the (cyclen·H^+^)_2_·Cl^−^ complex. For this purpose, we employed a classical trajectory method adapted from previous studies (Mesleh et al., 1996; Campuzano et al., 2012), in which the atoms in the molecular system are treated as individual scattering centers. Within this approach, each atom interacts with N_2_ through short-range van der Waals forces and longer-range charge-induced dipole forces. The effective charges for the atoms were adopted from the natural charges derived from the MP2 computation. This methodology was successfully applied in recent ion mobility investigations in our group, involving protonated monomers, dimers, and trimers of alcohols, cetones, and aldehydes of different size (Jurado-Campos et al., 2018).

The theoretical IR spectrum of each conformer was produced by convoluting the normal modes of vibration obtained in the MP2 computation with a line broadening of 25 cm^−1^ (full width at half maximum) and a scaling of the MP2 harmonic vibrational frequencies by a factor 0.97. It will be shown that the agreement of the computational IR spectra with the IRMPD measurements demands consideration of anharmonic behavior. Anharmonic corrections of the fundamental vibrational modes of the two most stable conformers of the complex were computed at the B3LYP-D3/6-311++G(d,p) level, with the generalized second-order vibrational perturbation method (GVPT2) (Barone et al., 2012; Bloino et al., 2012), as implemented in Gaussian 09 (Frisch et al., 2009). A full anharmonic computation was not possible with our computational resources. Therefore, restricted mode computations (Barone et al., 2012) were performed in which the anharmonic treatment was applied exclusively to selected ensembles of fundamental modes involving motions of the protonated -${\text{NH}}_{2}^{+}$ moiety, following a similar strategy as the one applied in a previous study for protonated cylen (Avilés-Moreno et al., 2018). The harmonic B3LYP-D3 vibrational frequencies were scaled by a factor 0.985. No scaling factor was applied to the computed anharmonic frequencies.

## 3. Results

### 3.1. Binary Complex (Cyclen·H^+^)·Cl^−^

A previous study in our group served to characterize the most salient structural features of isolated protonated cyclen (Avilés-Moreno et al., 2018). It was found that a strong proton bond is formed between two nitrogen atoms across the cyclen cavity. As a result, the vibrational modes of the macrocycle are severely perturbed, posing a serious challenge to the accurate description of the system. The initial issue that we try to elucidate in this work is to what extent the binding of the chloride anion alters the structure of protonated cyclen. The charge redistribution that accompanies NH^δ+^···Cl^−^ bonding can be expected to induce changes in the structure of the cyclen substrate and the strength of the intracavity proton bond.

**Figure 1:**
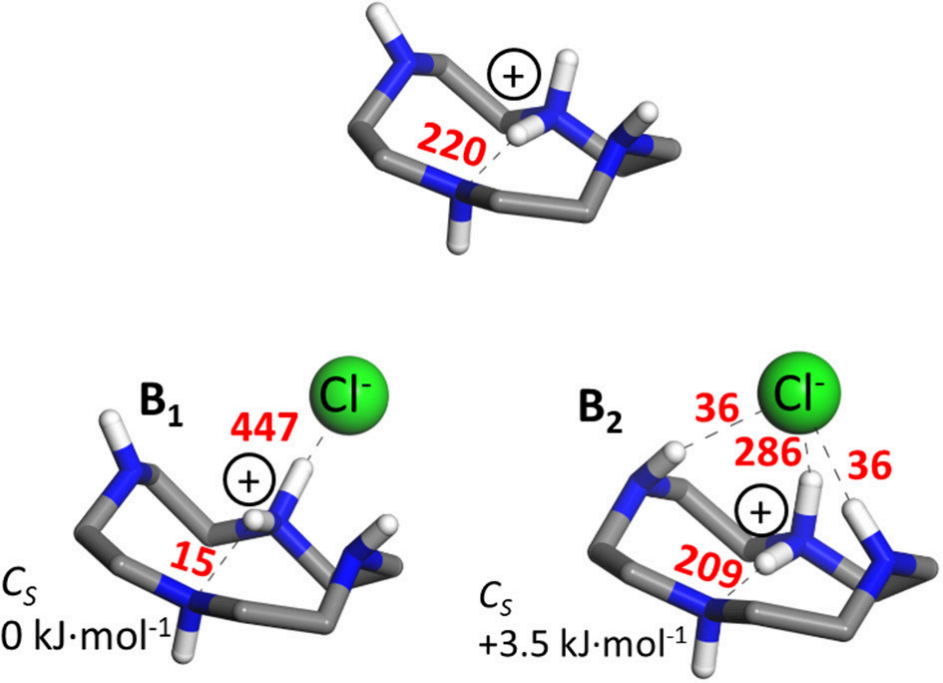
**(Top)** Most stable conformation of isolated protonated cyclen (Avilés-Moreno et al., 2018). **(Bottom)** Two most stable conformers of the binary complex of protonated cyclen with Cl^−^ found in this study [**B**_1_ and **B**_2_, Δ*E*_*zp*_= +3.5 kJ·mol^−1^ at MP2/6-311++G(d,p) level of theory]. Cl, N, C, and H are represented in green, blue, gray, and white color, respectively. The H atoms from the CH_2_ groups of cyclen have been removed for a better visualization of the structures (all three of *C*_*s*_ symmetry). The stabilization energies from NBO analysis, associated with the most relevant intermolecular NH^δ+^···Cl^−^/NH···Cl^−^ and intramolecular NH^δ+^···N interactions are indicated (in kJ·mol^−1^).

Our MP2/6-311++G(d,p) computations for the binary cyclenH^+^·Cl^−^ ion pair complex led to the two most stable configurations **B_1_** and **B_2_**, depicted in Figure 1. In the conformation of lowest energy, **B_1_**, chloride binding induces a reorientation of the ${\text{NH}}_{2}^{+}$ group in cyclen, leading to a NH^δ+^···N bond angle of 132^o^ and a proton bond distance of 2.4 Å across the cavity, in comparison to 161^o^ and 1.7 Å in isolated protonated cyclen. Consequently, the intramolecular proton bond is disrupted and the associated stabilization energy, as determined from NBO analysis, decreases by one order of magnitude (15 vs. 220 kJ·mol^−1^), whereas the proton-chloride bond is stabilized by as much as 447 kJ·mol^−1^. In the conformation next in energy for the binary complex, **B_2_**, the chloride anion occupies a more centered position above the cyclen cavity where it benefits from additional H-bonding with two inward-oriented neutral NH groups in addition to the proton-chloride bond. The distortion of the internal proton bonding in the macrocycle is in this case less appreciable than in conformer **B_1_**; the NH^δ+^···N bond angle and the proton bond distance are kept at values similar to isolated protonated cyclen, namely 170^o^ and 1.8 Å, respectively. The NBO stabilization energy of the intracavity proton bond in **B_2_** stays at a value of 209 kJ·mol^−1^, similar to that of the isolated macrocycle. The corresponding energy of the NH^δ+^···Cl^−^ bond is large (286 kJ·mol^−1^), but it diminishes appreciably with respect to **B_1_**. Finally, the contribution of the lateral NH···Cl^−^ bonds is significant but comparably more moderate, as it amounts to 36 kJ·mol^−1^ per bond.

In summary, the MP2 computation predicts two conformations relatively close in energy for the binary complex (cyclen·H^+^)·Cl^−^. The internal structure of the two conformations differ in qualitative aspects, such as the relative position of the Cl^−^ anion, the orientation of the neutral NH bonds of the macrocycle and the strength of the intracavity proton bond. Both conformers constitute plausible building blocks of the (cyclen·H^+^)_2_·Cl^−^ complex object of the present study. In fact, it is shown below that binary subunits resembling **B_1_** and **B_2_** are present in most of the low energy arrangements obtained independently for the ternary complex.

### 3.2. Ternary Complex (Cyclen·H^+^)_2_·Cl^−^

**Figure 2:**
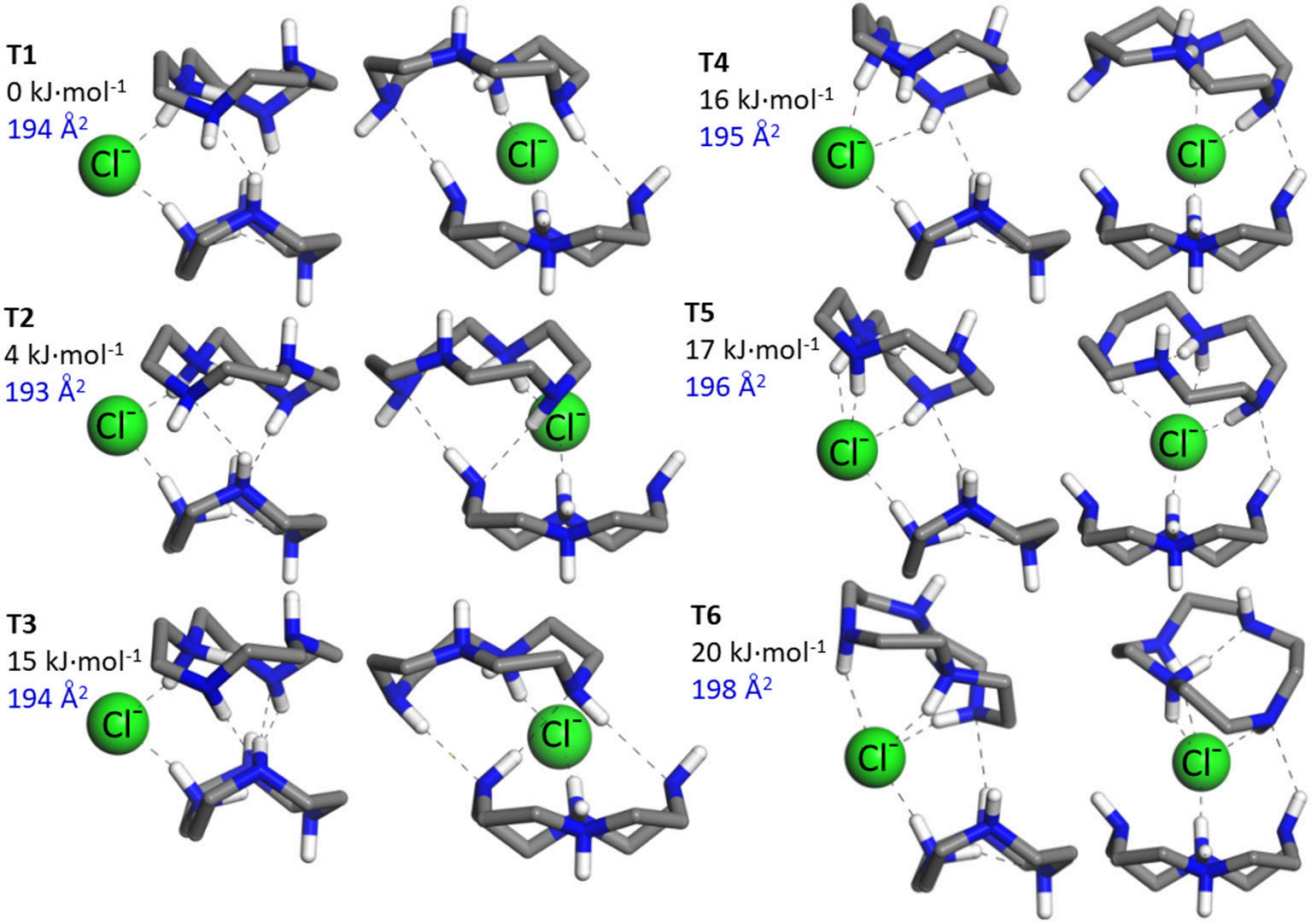
The six most stable conformers, **T_1_**–**T_6_**, of the ternary complex formed by two protonated cyclen units with Cl^−^ derived from our MP2/6-311++G(d,p) computations. The H atoms from the CH_2_ groups of cyclen have been removed for a better visualization of the structures. All the conformations correspond to folded arrangements, in most of which the binary conformations *B*_1_ and *B*_2_ depicted in Figure 1 can be identified with slight variations as building units. The relative energy (Δ*E*_*zp*_, in kJ·mol^−1^) and the N_2_ collision cross section (in Å) associated with each conformer are indicated.

**Figure 3:**
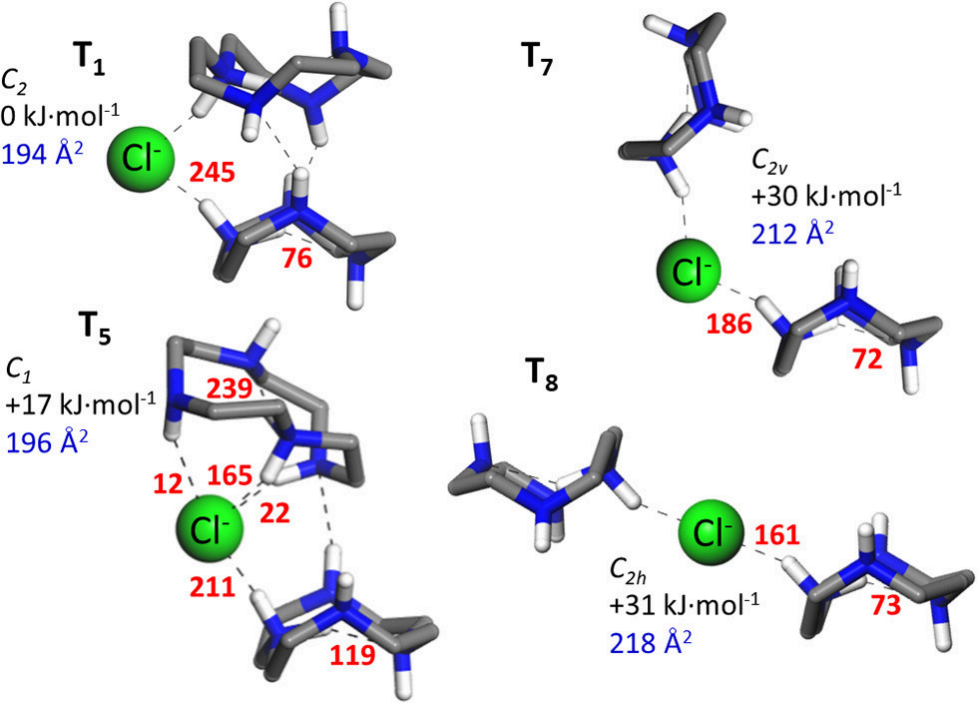
Illustrative low-energy MP2/6-311++G(d,p) conformations of the ternary complex formed by two protonated cyclen units with Cl^−^. **T_1_** and **T_5_** represent characteristic folded arrangement, whereas **T_7_** and **T_8_** constitute the open chain-line conformers of lowest energy found in our survey. The H atoms from the CH_2_ groups of cyclen have been removed for a better visualization of the structures. Next to each conformer, the point symmetry of the structure, the relative energy (Δ*E*_*zp*_) and the N_2_ collision cross section relevant to this study are indicated. In addition, NBO stabilization energies associated with the most relevant intermolecular and intramolecular interactions are indicated next to each non-covalent bond (redundant values due to symmetry are obviated).

An ensemble of prototypical low energy folded and open configurations of the (cyclenH^+^)_2_·Cl^−^ complex predicted by the MP2 computation is depicted in Figures 2, 3. The six conformations of lowest energy, **T_1_**–**T_6_**, correspond to folded arrangements stabilized by H-bonding interactions between the cyclen backbones in addition to the NH^δ+^···Cl^−^ bonds. Open chain-like conformations, with negligible interactions between the two cyclen hosts were also found in our survey. The most stable of these conformations, **T_7_** and **T_8_** (Figure 3), lie *ca*. 30 kJ·mol^−1^ higher in energy than the lowest energy folded conformer **T_1_**. Despite such high relative energy, the stretched configurations may be entropically favored by the rotational freedom of the two cyclen macrocycles around the chloride bonds. It is therefore not necessarily straightforward to draw predictions about the balance of the net populations of the folded vs. stretched conformational subsets.

We performed ion mobility measurements to assess the overall configuration of the (cyclen·H^+^)_2_·Cl^−^ complex, seeking to elucidate whether open or folded arrangements are dominant under isolated conditions. Ion mobility mass spectrometry allows to discern between conformations with a significant difference in effective size. Clearly, the compact folded arrangement in conformers **T_1_**–**T_6_** must have a smaller ”collisional” size in comparison to the stretched conformers **T_8_** and **T_9_**. The experiments yielded a room temperature N_2_ collision cross section of 198 ± 2 Å^2^, where the 1% uncertainty is associated with the dispersion of the results obtained in two separate equipments, as mentioned in section 2. The ion mobility spectra displayed a single drift peak with no trace of additional peaks that could be interpreted in terms of the simultaneous presence of complexes in conformational subsets with substantial differences in collision cross section (the full-width at half maximum of the drift peak accounts to less than 3 Å^2^ in cross section).

The N_2_ collision cross sections obtained in the classical trajectory calculation are indicated next to the corresponding conformers in Figures 2, 3. The simulation predicts cross sections within 193–198 Å^2^ for the folded conformers **T_1_**–**T_6_**, and consistently larger values of 212 and 218 Å^2^ for **T_7_** and **T_8_**, respectively. The statistical error of the simulations is of less than ±1 Å^2^. A greater source of uncertainty in these simulations arises from the choice of partial charges assumed for the atoms of the system. The NBO natural charge model employed in this study is recognized as a sensible framework with little sensitivity to the choice of basis set (Mao, 2014). Nevertheless, other choices are possible, which may have a sizeable impact on the estimated cross section. Test simulations with Mulliken partial charges for the present system led to cross sections within 2% of the values obtained with NBO charges (i.e., deviations were in all cases smaller than 4 Å^2^). Differences of similar magnitude have been reported in previous ion mobility studies of other ions (Lee et al., 2018). In the light of these results, it can be concluded that the ion mobility measurements support the greater stability of the folded conformations of the (cyclenH^+^)_2_·Cl^−^ complex (represented by **T_1_**–**T_6_**), under the present isolated conditions.

Figure 2 shows that in the folded arrangements, the two macrocyles are linked through NH^δ+^···Cl^−^···^δ+^HN bonds, in which the anion virtually acts as a flexible hinge that allows different types of relative orientations and H-bonding interactions between the cyclen backbones. Note that the **B_1_** and **B_2_** conformers of the (cyclen·H^+^)·Cl^−^ ion pair complex can be recognized with slight variations as building blocks of the ternary complexes. **B_1_** can be identified in conformers **T_1_**–**T_4_**, although with an inward reorientation of one of the NH bonds in some cases. The higher lying conformers, **T_5_** and **T_6_**, can be visualized as combinations of **B_1_** and **B_2_** subunits. Interestingly, the stabilization energies associated with the intermolecular proton-halide and the intramolecular proton-nitrogen bonds characteristic of the binary conformations **B_1_** and **B_2_** are qualitatively conserved in the ternary conformers. This finding is illustrated in Figure 3 for selected configurations. It can be appreciated that in **T_1_** strong NH^δ+^···Cl^−^ bonds are formed, at the cost of a significant weakening of the intracavity proton bond of the cyclen macrocycles, in the same way as found for the **B_1_** conformer. Incidentally, it is also shown that these same features are present in the open conformations **T_7_** and **T_8_**, which can actually be considered stretched counterparts of conformer **T_1_**. Finally, inspection of the stabilization energies in conformer **T_5_** reveals that its subunit analogous to **B_2_** maintains a strong intramolecular proton bond and a somewhat weaker proton bond with Cl^−^ than its other **B_1_**–like subunit.

**Figure 4:**
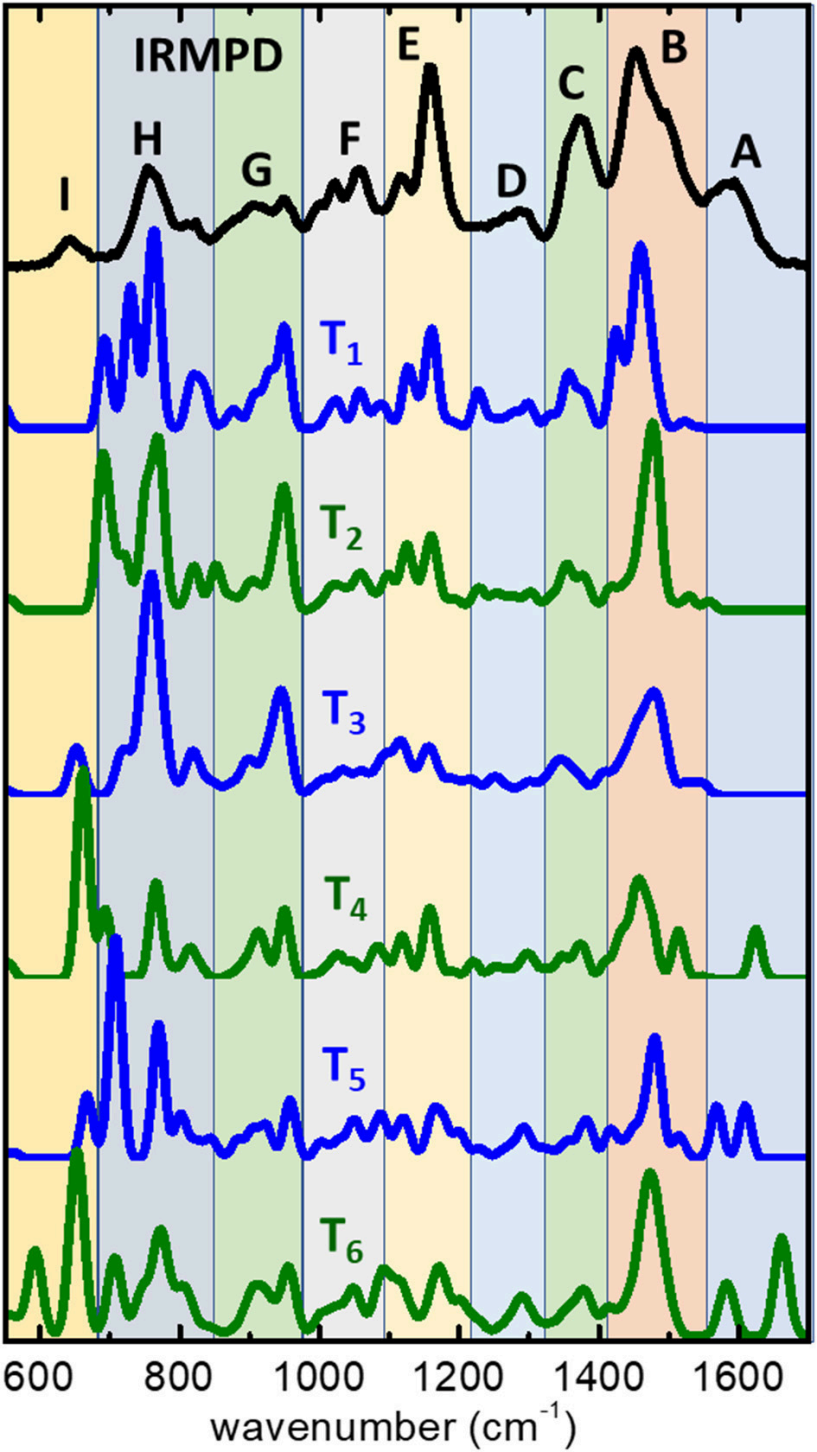
IRMPD spectrum measured for the (cyclen·H^+^)_2_·Cl^−^ complex ion (upper trace), and harmonic spectra predicted by the MP2/6-311++G(d,p) computation for the low energy conformers **T_1_**–**T_6_** (see Figure 2). An assignment for the main bands observed experimentally (labeled A–I) is provided in Table 1, in terms of the dominant type of vibrational motions involved in each spectral region.

The proton-bonding and H-bonding arrangements predicted by the MP2 computations can be expected to lead to differentiated spectral signatures amenable of being discerned experimentally. The IRMPD spectrum measured for the (cyclen·H^+^)_2_·Cl^−^ complex is displayed in Figure 4, where it is compared to the computational IR spectra associated with the low energy conformers **T_1_**–**T_6_**. The IRMPD spectrum displays a complex progression of partially overlapping bands of varying intensity within the spectral window covered by the present experiments. The MP2 computation reproduces correctly most of the features of the observed IRMPD bands, which facilitates the interpretation of the spectrum. Table 1 provides a qualitative assignment of the main bands, based on the dominant vibrational motions predicted by the computation in each spectral region. Importantly, the fundamental modes more closely associated to bending motions of the charged ${\text{NH}}_{2}^{+}$ moieties are located on the high frequency region (scissoring, wagging, band A) and on the low frequency region (rocking, bands G and I) of the recorded spectrum. The central part of the spectral range (1,000–1,500 cm^−1^) displays vibrational motions of the remaining groups of the cyclen backbone (C-C and C-N stretching, NH and CH_2_ bending vibrations).

**Table 1:** Assignment for the main bands observed in the IRMPD spectrum of the (cyclen·H^+^)_2_·Cl^−^ complex, based on the dominant type of vibrational motions predicted by the MP2/6-311++G** computation (see labels in Figure 4).

**Band**	**Mode assignment**
A	${\text{NH}}_{2}^{+}$ scissoring and wagging
B	CH_2_ scissoring, NH wagging
C	CH_2_ wagging
D	CH_2_ twisting
E	CN stretching
F	CC and CN stretching
G	CC stretching, ${\text{NH}}_{2}^{+}$ rocking
H	CH_2_ and NH rocking
I	${\text{NH}}_{2}^{+}$ and NH rocking

The MP2 infrared spectrum for the lowest energy conformer **T_1_** resembles nicely the structure of the IRMPD measurement over most part of the spectral range. The most remarkable difference is related to the apparent absence of band A in the MP2 spectrum. Bands B through F are reproduced fairly well by the computation, despite some differences in their relative intensities. A partial matching is found for bands G, H, and I, associated with rocking vibrations of the ${\text{NH}}_{2}^{+}$ and NH groups, although in these cases significant discrepancies in shape and relative intensities are found between computation and experiment. In particular, the MP2 computation predicts strong band components in the IR spectrum of **T_1_** within 680–800 cm^−1^, which overestimate the relative yield measured for band H and may contain contributions actually related to band I, for which no clear trace is found in the MP2 spectrum. The MP2 spectra of the higher lying conformers, **T_2_**–**T_6_**, display similar discrepancies with experiment for bands H and I, although the agreement improves in the case of **T_3_**.

**Figure 5:**
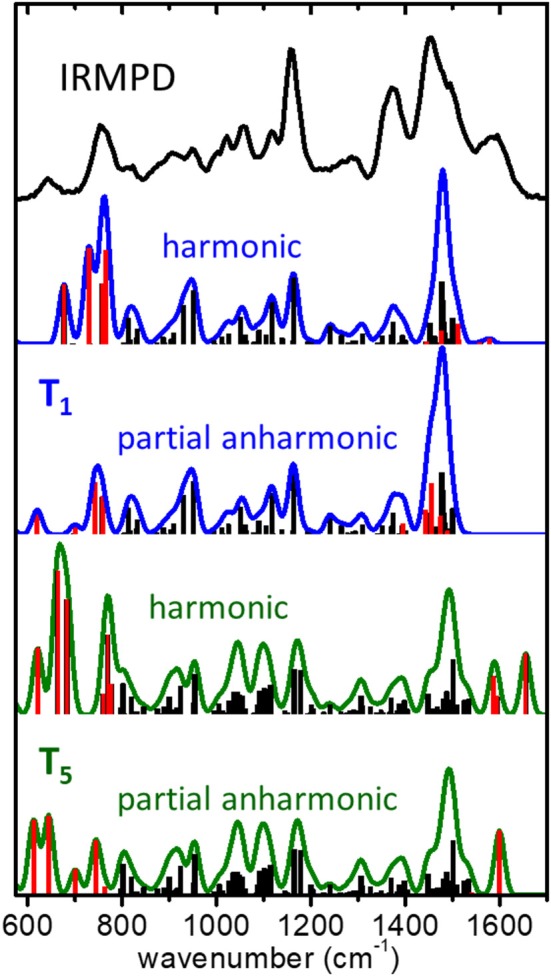
IRMPD spectrum measured for the (cyclen·H^+^)_2_·Cl^−^ complex ion, compared to harmonic and partial anharmonic IR spectra predicted by the B3LYP-D3/6-311++G(d,p) computation for the low energy conformers **T_1_** and **T_5_**. The partial anharmonic computations applies the GVPT2 approximation to the fundamental modes directly related to vibrational motions of the ${\text{NH}}_{2}^{+}$ protonated moiety of cyclen (modes represented in red color in the bar diagrams).

The MP2 spectrum of conformers **T_4_**, **T_5_**, and **T_6_** do show qualitative differences with respect to **T_1_**–**T_3_** in the high frequency range. The main novel feature is the presence of intense vibrational transitions for the scissoring bending modes of the ${\text{NH}}_{2}^{+}$ protonated group at frequencies above 1,500 cm^−1^. This result is remarkable, as it serves to rationalize the presence of band A in the IRMPD spectrum. Double peak structures are found with different positions and relative spacings for each of the conformers. The best agreement is found for **T_5_** which displays transitions in the range 1,560–1,620 cm^−1^, which is coincident with the envelope of the experimental band A. The analogous scissoring transitions predicted for the **T_1_** conformer appear at lower frequencies, ~ 1,450–1,520 cm^−1^, overlapping in frequency with the CH_2_ scissoring and NH wagging transitions that conform band B in the MP2 spectrum. This aspect is appreciated in Figure 5 (red histograms), as discussed below in detail. It becomes apparent from the result of the MP2 computations, that the fundamental modes of the -${\text{NH}}_{2}^{+}$ moiety are particularly sensitive to the conformational subtleties of the halide coordination arrangements.

Despite the discrepancies described above, the fairly good overall agreement found between the IRMPD measurement and the computational IR spectra, validates the low energy landscape of the complex described by the conformations depicted in Figure 2. Conformational arrangements of the types represented by **T_1_**–**T_6_** plausibly coexist at room temperature under the present experimental conditions. The joint contribution of various types of folded conformers seems to be required for an appropriate reproduction of the most salient features of the IRMPD spectrum. Whereas, the MP2 spectra of the six conformers reproduce most of the observed bands, **T_5_** and similar conformations improve the agreement and are definitely required to account for band A. In addition, band B of the IRMPD spectrum displays a broadened structure, with a hint of a non-resolved shoulder on its blue flank, that is well-accounted for by the joint contributions of the several conformers.

Our previous IRMPD study of protonated cyclen suggested that anharmonic behavior must be taken into account for the accurate description of the vibrational spectrum (Avilés-Moreno et al., 2018). Therefore, it seemed timely to incorporate anharmonicity to the modeling of the (cyclen·H^+^)_2_·Cl^−^ complex, seeking to improve the comparison of the computational and experimental spectra. The focus was in the regions below 800 cm^−1^ and above 1,400 cm^−1^ where the most appreciable differences are found. These spectral regions contain fundamental modes involving vibrational motions of the ${\text{NH}}_{2}^{+}$ group (see Table 1). The most intense of those modes at a harmonic level were selected for the anharmonic treatment, while the rest of modes were kept within the harmonic approximation.

Figure 5 illustrates the resulting harmonic and partial anharmonic spectra obtained at the B3LYP-D3/6-311++G** level for conformers **T_1_** and **T_5_**. A histogram representation of the fundamental frequencies is included, in which the modes chosen for the anharmonic treatment are highlighted in red color for a better visualization of the changes induced by anharmonicity. A total of 12 and 9 fundamental modes were included in the anharmonic computations for **T_1_** and **T_5_**, respectively. It is interesting to find that the incorporation of the anharmonic corrections brings the computational spectra to a significant better agreement with the IRMPD measurement. For the **T_1_** conformer, the anharmonic computation reproduces quite accurately the position and relative intensities of bands H and I. The effect in the high frequency range of the spectral window is less noticeable, as the anharmonic modes shift slightly with respect to their harmonic counterparts but stay within the envelopes of bands B and C. For the **T_5_** conformer, the agreement for bands H and I also improves appreciably. In this case, the most remarkable finding is related to band A, for which the anharmonic computation predicts a single dominant vibrational transition at ~1,600 cm^−1^ (as opposed to the bimodal peak structure of the harmonic computation), in excellent agreement with the IRMPD measurement.

The present incursion into the anharmonic behavior of the isolated (cyclen·H^+^)_2_·Cl^−^ complex provides a more solid qualitative and quantitative support to the conclusion, anticipated from the harmonic computations, that a joint contribution of folded conformations of the **T_1_** and **T_5_** types is required to reproduce the main signatures of the IRMPD spectrum. Hence, the room temperature conformational landscape of the complex can be considered to be well-represented by the ensemble of low energy structures compiled in Figure 2.

## 4. Summary and Concluding Remarks

A combination of action IRMPD spectroscopy and ion mobility mass spectrometry with quantum chemical computations has served to elucidate the preferential conformations and coordination arrangements in the isolated supramolecular system comprised by two protonated cyclen macrocycles linked by a chloride anion.

The Cl^−^ anion bridges the protonated ${\text{NH}}_{2}^{+}$ moieties of the two macrocycles, leading to a molecular tweezer configuration. The IRMPD experiments suggests that various types of folded conformations coexist at room temperature, featuring either peripheral or inner positions of the anion with respect to the macrocycle cavities and H-bonds between the cyclen backbones (Figure 2). Open chain–like configurations (Figure 3) lay appreciably higher in energy according to the MP2 computations, and their significant population at room temperature is also ruled out by the ion mobility measurements.

The ${\text{NH}}_{2}^{+}$-halide bond shows robustness and flexibility as to provide for a varied landscape of coordination structures with azamacrocycles. The opening and folding of the (cyclen·H^+^)_2_·Cl^−^ complex in condensed phase can be expected to be modulated by the interactions with neighboring species and solvent molecules, plausibly leading to sensing or caging properties, as well as serving as seeding substrates for the growth of extended networks through additional protonation and incorporation of anions.

The modeling of these materials presents however important challenges, due to the multiple intermolecular and intramolecular proton bonding potentially involved. This study has shown that the proton interactions characteristic of isolated protonated cyclen and of the binary (cyclen·H^+^)·Cl^−^ complex, are to a large extent retained in the (cyclen·H^+^)_2_·Cl^−^ complex, although with significant differences among the low-energy conformations. In particular, the strength of the intramolecular NH^+^···N proton bond in the cyclen macrocycle and of the intermolecular ${\text{NH}}_{2}^{+}$···Cl^−^ bonds are sensitive to fine details of the coordination geometry and the orientation of the charged moiety with respect to the macrocycle cavity. A complex, plausibly dynamic, picture of halide bonding in azamacrocycles emerges, taking into account that a variety of conformers are likely to be populated at room temperature. Whereas, the overall conformations of the complexes may be captured at a moderate level of theory, the assessment of electronic structure, bond strengths, and the related spectroscopic features is demanding. This study has served to illustrate that the accurate description of the vibrational features of polyazamacrocycles requires an anharmonic treatment of the protonated -${\text{NH}}_{2}^{+}$ moieties. Importantly, the application of partial schemes to treat anharmonicity, restricted to specific ensembles of fundamental modes, has been shown to provide a fair approximation to the vibrational spectrum over a broad frequency region. This should be relevant in particular if spectroscopic signatures are to be employed for the elucidation of the coordination structures achieved between the azamacrocycle and the halide anions. We expect that the fundamental insights laid out in this study constitute a valuable benchmark to guide the modeling and characterization of this class of materials.

## Data Availability

The datasets generated for this study are available on request to the corresponding author.

## Author Contributions

All authors listed have made a substantial, direct and intellectual contribution to the work, and approved it for publication.

### Conflict of Interest Statement

The authors declare that the research was conducted in the absence of any commercial or financial relationships that could be construed as a potential conflict of interest.

## Footnotes

^1^http://www.ru.nl/felix/
